# Preliminary Psychometric Validation of the Teammate Burnout Questionnaire

**DOI:** 10.3389/fpsyg.2022.894308

**Published:** 2022-08-01

**Authors:** Ralph Appleby, Paul Anthony Davis, Louise Davis, Andreas Stenling, Will Vickery

**Affiliations:** ^1^The Talented Athlete Scholarship Scheme (TASS), Newcastle upon Tyne, United Kingdom; ^2^Department of Psychology, Umeå University, Umeå, Sweden; ^3^Coaching and Officiating, Sport Australia, Canberra, ACT, Australia

**Keywords:** exhaustion, social environment, social perceptions, measurement, team sport

## Abstract

The aim of the present study was to provide support for the validation of the Teammate Burnout Questionnaire (TBQ). Athletes from a variety of team sports (*N* = 290) completed the TBQ and the Athlete Burnout Questionnaire (ABQ). Confirmatory factor analysis revealed acceptable fit indexes for the three-dimensional models (i.e., physical and emotional exhaustion, sport devaluation, reduced accomplishment) of the TBQ and the ABQ. Multi-trait multi-method analysis revealed that the TBQ and ABQ showed acceptable convergent and discriminant validity. The preliminary validation of the TBQ indicates the utility of the scale to reflect athletes’ perceptions of their teammates’ burnout and offers researchers the opportunity to quantitatively assess an important aspect of the social environment in the development of athlete burnout.

## Introduction

Over the last 20 years the prevalence of athletes reporting burnout symptoms has increased (Madigan et al., 2022). Burnout is recognized as an indicator of an athlete’s maladaptation to psychosocial demands and is associated with a variety of negative outcomes such as decreased well-being and reduced performance (DeFreese and Smith, 2013; Madigan et al., 2016; Gustafsson et al., 2017; Smith et al., 2019). The negative consequences of burnout can amplify difficulties with motivation and can lead to dropout from sport (Larson et al., 2019). Athlete burnout is generally defined as a cognitive-affective syndrome comprised of physical and emotional exhaustion, reduced sporting accomplishment, and devaluation of sport participation (Raedeke and Smith, 2001). Specifically, *physical and emotional exhaustion* is characterized by the perceived depletion of emotional and physical resources as a consequence of training and/or competition. *Reduced sporting accomplishment* reflects an individual’s negative evaluation of sporting abilities and achievements. The *devaluation of sport participation* is defined as the diminishment of perceived benefits of being involved in sport (Raedeke, 1997; Gustafsson et al., 2017). It is noted that the underlying aspects of exhaustion, reduced accomplishment, and devaluation can vary across athletes (Eklund and DeFreese, 2020). Despite a widespread acceptance of the conceptualization of athlete burnout, and multiple decades of research pursuing the established line of enquiry, the need for further research examining the role of social constructs in athletes’ perceptions of burnout has been highlighted (Pacewicz et al., 2019).

Although the precise etiology of athlete burnout is uncertain, athletes’ stress is associated with development of athlete burnout symptoms (Lin et al., 2021). Previous longitudinal research has found that raised levels of stress precede increases in athlete burnout (DeFreese and Smith, 2014). In particular, the multidimensional aspects of athlete burnout are noted to be influenced by the demands of physical training (e.g., excessive training; Gustafsson et al., 2011) as well as psycho-social stressors that are associated with sport (e.g., teammates; Pacewicz et al., 2020). Athletes’ perceptions of their social environment can manifest as psychophysiological symptoms associated with burnout (Barcza-Renner et al., 2016); considering athletes do not partake in sport in isolation, rather they engage with various social agents (e.g., coaches, teammates) within the sporting context, it is important to be able to measure the influence of significant others to advance understanding of the impact of social factors (Pacewicz et al., 2019). Supportive social interactions within the sporting environment have the potential to positively influence and enhance athletic performance (Bianco and Eklund, 2001). On the other hand, unwanted, rejecting or neglecting behaviors that typify negative social interactions (with social agents) can hinder progress and result in a negative athlete experience (Newsom et al., 2005).

Gustafsson et al.’s (2011) integrated model of athlete burnout incorporates aspects of the social environment within the proposed antecedents of burnout (e.g., stressful social relations) by building upon early research that suggests negative social interactions may compound the risk of burnout (e.g., Cresswell and Eklund, 2006a). Previous qualitative research (e.g., Cresswell and Eklund, 2006b,^2007^) highlights that a negative team atmosphere consisting of dishonesty and a lack of trust in management as well as coach pressure, increases the likelihood of athletes developing burnout. Furthermore, related research investigating cohesion and burnout in teams has noted that antisocial behavior positively predicts perceptions of athlete burnout (Al-Yaaribi and Kavussanu, 2017).

Taken collectively, research suggests an athlete’s social environment can influence perceptions of burnout through antecedents as well as protective mechanisms, however, specific social constructs require further empirical study (Pacewicz et al., 2019). It has been proposed that athlete burnout can manifest itself behaviorally as well as socially (Schaufeli and Enzmann, 1998; Eklund and DeFreese, 2020); yet, research has predominantly investigated burnout at the individual level with limited measurement of social factors (Pacewicz et al., 2019). More recently, there has been increasing interest regarding the influence of social constructs such as group dynamics (e.g., cohesion) on athletes’ perceived motivation and burnout (Pacewicz et al., 2020). In an examination of team-sport athletes’ levels of burnout, Appleby et al. (2018) observed that an athlete’s level of burnout was associated with perceptions of their teammates’ burnout. One potential explanation for this finding was that athletes interpret perceptions of their own burnout in relation to the team environment, and subsequently associate evaluations of their own burnout with their teammates’ as a consequence of the shared experience (i.e., number of training hours). As such, a validated measure of athletes’ perceptions of their teammates’ burnout appears to be warranted in order to advance understanding of the role of social factors in athlete burnout.

The Athlete Burnout Questionnaire (ABQ; Raedeke, 1997; Raedeke and Smith, 2001, 2009) is the most commonly used method of assessing athlete burnout in sport psychology research and applied practice (Gustafsson et al., 2014; Madigan et al., 2019) as it has been shown to possess good psychometric indictors of reliability (e.g., test-retest, internal consistency) and validity. Specifically, Multitrait-multimethod (MTMM) analyses evaluating the factorial, discriminant, and convergent validity of the ABQ and Maslach Burnout Inventory-General Survey (MBI-GS; Maslach et al., 2001) displayed acceptable convergent validity with highly correlated matching subscales and satisfactory internal discriminant validity with lower correlations between non-matching subscales (Cresswell and Eklund, 2006a). Furthermore, confirmatory factor analyses (CFAs) supported the theoretical subscales of the ABQ achieved through items adequately loading on the appropriate factors (Raedeke and Smith, 2001; Raedeke et al., 2013). Researchers have provided evidence for acceptable test-retest reliability of the ABQ across a 1–3-week period (Raedeke and Smith, 2001; Arce et al., 2012). In review of the modification of the MBI-GS to measure perceptions of colleagues’ burnout in other domains (e.g., Bakker and Schaufeli, 2000), the ABQ has previously been determined to be a reliable measure of athlete burnout and as such can be considered an appropriate basis for an adapted questionnaire to measure an athlete’s perception of teammates’ burnout.

In summary, there is limited understanding of the impact of an athlete’s contextual sporting environment and social interactions on the potential manifestation of burnout. As such, the influence of perceptions of teammates’ burnout and how this may influence an athlete’s own level of burnout warrants investigation. However, a validated measure of athletes’ perceptions of their teammates’ burnout is currently unavailable. The development of an instrument measuring burnout at a team level would support the examination of social factors as antecedents of burnout (Pacewicz et al., 2019), and the proposed impact of psychosocial stressors outlined in the Integrated Model of Athlete Burnout (Gustafsson et al., 2011).

The aim of the present study was to validate a three-factor Teammate Burnout Questionnaire (TBQ) comprised of subscales reflecting teammates’ sport devaluation, teammates’ emotional and physical exhaustion, and teammates’ reduced accomplishment at the team level. To do so, CFA and MTMM analyses were chosen to assess the factorial validity, discriminant validity, and convergent validity of the TBQ and ABQ. The hypotheses for the modeling were that the TBQ and ABQ would demonstrate factorial validity, and the TBQ would demonstrate discriminant and convergent validity.

## Methods

### Participants

A total of 290 athletes, including 170 males (58.6%) and 120 females (41.4%), participated in the study. The participants ranged in age from 18 to 35 years, with a mean age of 20.97 years (*SD* = 3.08). All of the athletes played team-sports, representing eight different popular sports in the United Kingdom: football (*n* = 44, 15.2%), netball (*n* = 21, 7.2%), rugby (*n* = 33, 23.6%), Gaelic football (*n* = 15, 5.2%), cheerleading (*n* = 28, 9.7%), volleyball (*n* = 34, 11.7%), rugby league (*n* = 19, 6.6%), and field hockey (*n* = 23, 7.9%). All participants were members of teams currently undertaking inter-team competitions, ranging from regional to professional. The participants trained with their teammates between 1 and 3 times a week for an average of 8.65 h (*SD* = 4.45) and reported to have played together for an average 2.46 years (*SD* = 2.57). Data collection occurred during the competitive season.

### Measures

#### Demographic and Background Inventory

Participants reported a variety of demographic information including: age, gender, how often they train together as a team, and years played with current team.

#### Athlete Burnout

Each athlete’s level of burnout was assessed using the ABQ (Raedeke and Smith, 2001). This 15-item self-report measure is comprised of questions which assess the subscales of physical and emotional exhaustion (e.g., “I feel overly tired from my sport participation”), reduced accomplishment (e.g., “I am not performing up to my ability in sport”), and sport devaluation (e.g., “I don’t care as much about my sport performance as I used to”). Each of the subscales are measured with the five items, and the stem for each was “How often do you feel this way?” to which participants responded on a five-point Likert Scale anchored by (1) “*Almost Never*” and (5) “*Almost Always*”. Previous research has supported the validity and reliability of the ABQ, factor structure, and internal consistency (α ≥ 0.85; Raedeke and Smith, 2001, 2009). Within this study the ABQ showed good psychometric properties with acceptable internal consistencies (α > 0.75) for all three of the subscales. Scores reflecting each of the subscales were calculated by determining the mean of the associated items and a global athlete burnout score was calculated by averaging the scores of the 15 items comprising the ABQ.

#### Teammate Burnout

The Teammate Burnout Questionnaire (TBQ) was developed in line with the referent-shift consensus model (Chan, 1998). That is, the conceptual definition of athlete burnout was adapted to reflect a higher level within-group aggregated construct of teammate burnout. Specifically, the items of the TBQ were adapted from the ABQ (Raedeke and Smith, 2001) to reflect the perception of the individual about his or her teammates’ burnout symptoms. The TBQ is a 15-item self-report measure that is comprised of questions that assess the subscales of teammate physical and emotional exhaustion (e.g., “My teammates feel overly tired from their sport participation”), teammate reduced accomplishment (e.g., “My teammates are not performing up to their ability in sport”), and teammate sport devaluation (e.g., “My teammates don’t care as much about their sport performance as they used to”). The subscales are each measured with the use of five items, and the stem for each was “How often do your teammates feel this way?” to which participants responded, on a five-point Likert Scale anchored by (1) *“Almost Never”* and (5) *“Almost Always”.* Data analysis of the sample in the present study indicate the TBQ showed good psychometric properties with acceptable internal consistencies (α > 0.75) for all three subscales. Previous research has reported good internal consistency (α ≥ 0.80) for each of the three subscales of the TBQ (Appleby et al., 2018). Scores reflecting each of the subscales were calculated by determining the mean of the associated items and a global teammate burnout score was calculated by averaging the scores of the 15 items comprising the TBQ.

### Procedure

Ethical approval was granted from the research ethics committee of the first author’s university prior to conducting the study (RE-HLS-20112014). In order to recruit participants, the directors of sports clubs and head coaches of sports teams were contacted via e-mail and follow-up phone calls where necessary, to obtain permission to conduct the study at their respective organizations. Following the consent from directors and coaches, the first author attended a training session to outline the aims and objectives of the study to a group of athletes and to gain athletes’ consent. Information sheets outlining the aims of the study were then provided to the athletes prior to participating and written consent was obtained. Participants were reassured of confidentiality and told their data would be assigned a randomized participation number to maintain anonymity. Data were collected prior to the commencement of a training session around the mid-stages of the competitive season. The aim of the data collection process was designed to assess the preliminary validity of the TBQ; therefore participants were provided with a multi-section questionnaire that consisted of questions pertaining to demographic information (e.g., age, gender, number of months/years playing with teammates), the ABQ, and the TBQ. This process required a maximum of 15 mins to complete, the first author was present during data collection and available to answer any queries. The participant written consent forms and participation numbers were securely kept separate to the data collected.

### Statistical Analysis

The analysis process had two stages executed in sequential order. First, confirmatory factor analysis of the ABQ and TBQ were conducted to evaluate the factorial validity of the questionnaires. Second, multi-trait multi-method analysis comprised of the ABQ and the TBQ were performed to assess the discriminant validity and convergent validity of the questionnaires. Full details of the process are included in the “Results” section below.

## Results

### Descriptive Statistics

Descriptive statistics and bivariate correlations were performed using SPSS version 22. Table 1 presents means, standard deviations, and bivariate correlations of all variables under investigation. Athletes’ scores on the subscales of the ABQ and the TBQ were relatively low, which is consistent with findings commonly reported in related literature (Raedeke and Smith, 2009; Gustafsson et al., 2015; Appleby et al., 2018). Pearson’s correlation co-efficients indicate that the three subscales of the TBQ were positively and significantly correlated (*r* = 0.358–0.703). The analysis showed positive and significant correlations between the three subscales of the ABQ (*r* = 0.242–0.530). The correlations between the ABQ and the TBQ subscales were positive and statistically significant (*r* = 0.198–0.648), refer to Table 1 for correlation values.

**TABLE 1:** Descriptive statistics and bivariate correlations for all main variables under investigation.

Variables	M	SD	1	2	3	4	5	6	7	8
**Athlete variables**										
(1) TRA	2.12	0.63								
(2) TE	2.54	0.71	0.358**							
(3) TSD	2.03	0.63	0.703**	0.506**						
(4) GTB	2.24	0.54	0.809**	0.771**	0.869**					
(5) RA	2.33	0.61	0.317**	0.244**	0.397**	0.382**				
(6) E	2.53	0.74	0.198**	0.648**	0.306**	0.491**	0.242**			
(7) SD	1.95	0.72	0.340**	0.265**	0.483**	0.430**	0.530**	0.349**		
(8) GAB	2.27	0.53	0.370**	0.518**	0.517**	0.573**	0.744**	0.719**	0.824**	

GTB, global teammate burnout; TRA, teammate reduced accomplishment; TE, teammate exhaustion; TSD, teammate sport devaluation; GAB, global athlete burnout; RA, reduced accomplishment; E, exhaustion; SD, sport devaluation. The symbol ** represents significance at 0.01.

### Confirmatory Factor Analysis

In order to evaluate the factorial validity of the questionnaires to assess athlete and teammate burnout the ABQ and the TBQ were analyzed using confirmatory factor analysis (CFA). The chi-square (χ^2^), comparative fit index (CFI), root mean square error of approximation (RMSEA) and its associated 90% confidence interval (RMSEA-CI), and Tucker-Lewis Index (TLI) were used to assess CFA model fit. Two CFA models were created using AMOS. Model A represents the ABQ encompassing all 15-items mapped on to the appropriate subscales (i.e., reduced accomplishment, exhaustion, and sport devaluation). Model B represents the TBQ including all 15-items corresponding to the subscales (i.e., teammate reduced accomplishment, teammate exhaustion, and teammate sport devaluation). The model fit criteria (i.e., χ^2^, CFI, TLI, and RMSEA) are outlined for each model in Table 2.

**TABLE 2:** Fit Indices on ABQ and TBQ.

						90% Cl	
Model	χ^2^	df	CFI	TLI	RMSEA	Lower	Upper
A	248.432	87	0.899	0.878	0.080	0.069	0.092
B	194.632	87	0.940	0.940	0.065	0.053	0.078

*χ^2^, Chi Square; df, degrees of freedom; CFI, Comparative Fit Index; TLI, Tucker Lewis Index; RMSEA, Root Mean Square Error of Approximation; Model A, CFA ABQ; Model B, CFA TBQ.*

### Multi-Trait Multi-Method Analysis

The next step was to combine both models into one MTMM analysis to test for discriminant validity and convergent validity. MTMM matrix level evaluation of construct validity involves the comparison of various nested models to determine convergent and discriminant validity (Byrne, 1994a).

**FIGURE 1:**
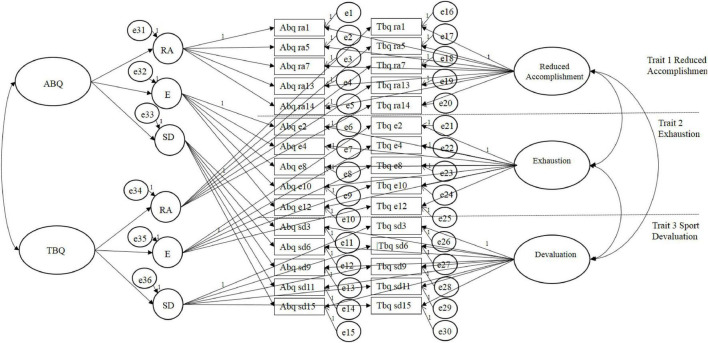
Hypothesized MMTM model (correlated traits – correlated methods).

Figure 1 illustrates the relationships between traits, methods, and the indicators underlying all the MTMM models analyzed in this study. Traits in Figure 1 represent the subscales of athlete burnout and teammate burnout, whereas the methods denote the questionnaires (i.e., ABQ or TBQ). The correlated traits-correlated method (CTCM) with second-order methods was chosen as the baseline model as athlete burnout comprises three subscales (i.e., reduced accomplishment, physical and emotional exhaustion, sport devaluation). Although exhaustion is considered to be the core dimension of burnout, many researchers argue that the other dimensions are required to capture the syndrome (Maslach et al., 2001; Gustafsson et al., 2011). This has theoretical implications for the MTMM modeling process as it lends itself to second-order method factors. In this proposed model, second-order factors (i.e., global scores on the ABQ and TBQ) represent the relations between first-order factors (e.g., exhaustion and teammate exhaustion); the first-order factors represent the relations between the corresponding items of each of the questionnaires.

The correlated traits-correlated method (CTCM) with second-order methods allows for a direct comparison between the ABQ and TBQ. Although, fully crossed MTMM models (all traits x all methods) evaluated using CFA often present inadmissible solutions and convergence problems (Marsh et al., 2002; Marsh, 2007), this approach was chosen due to the strong theoretical foundations and completeness of the model (Natesan and Aerts, 2016).

In the CTCM model all indicators were loaded uniquely upon trait (i.e., reduced accomplishment, exhaustion, and sport devaluation) and method (i.e., ABQ or TBQ). Trait and method factors were not allowed to correlate with one another. However, traits were allowed to correlate with other traits, and methods were allowed to correlate with other methods. In the subsequent models the loading of the indicators remains the same; it is the relationship between the traits and second order methods that are adjusted to allow for the comparison of the ABQ and TBQ. The other nested comparison models include: the correlated traits/uncorrelated methods model (CTUM; i.e., all traits are correlated freely and second ordered methods are uncorrelated), the correlate traits/perfectly correlated methods model (CTPCM; i.e., the model is specified by allowing the correlations between traits to vary and fixing the correlation between the second-order methods to (1), the perfectly correlated traits/correlated methods model (PCTCM; i.e., the correlation between traits are set to 1 and the correlation between methods is free to vary), the uncorrelated traits/correlated models (UTCM; i.e., no correlations between traits and methods are able to freely correlate), and the no traits/correlated methods model (NTCM; i.e., a model where traits are not included and methods are free to vary).

Values around 0.90 indicate acceptable fit for CFI and TLI, whereas values around 0.08 indicate acceptable fit for RMSEA (Marsh, 2007). Chi-square difference tests and Akaike’s information criterion (AIC where the lower score represent better fit; Buckland et al., 1997) were employed to statistically compare MTMM models to assess convergent and discriminant validity (Byrne, 1994a). The hypothesized model shown in Figure 1 has the same structure as the tested model in CTCM model presented in Table 3. All of the MMTM models converge appropriately. A summary of the models is presented in Table 4. All of the models exhibited significant χ^2^. Models with fewer degrees of freedom (i.e., CTCM and UTCM models) demonstrated acceptable fit. The fit of the PCTCM and NTCM were below the acceptable threshold. The poor fit of NTCM was anticipated as the model has no trait factors (Byrne, 1994a) and the poor fit of PCTCM was expected given that it essentially proposes a single trait factor (Cresswell and Eklund, 2006a).

**TABLE 3:** Method factor correlations.

Variables	1	2	3	4	5	6
(1) TRA	1					
(2) TE	0.442**	1				
(3) TSD	0.879**	0.583**	1			
(4) RA	0.369**	0.302**	0.491**	1		
(5) E	0.275**	0.750**	0.373**	0.349**	1	
(6) SD	0.430**	0.286**	0.592**	0.667**	0.409**	1

*TRA, teammate reduced accomplishment; TE, teammate exhaustion; TSD, teammate sport devaluation; RA, reduced accomplishment; E, exhaustion; SD, sport devaluation.*

**p significant at 0.01.

**TABLE 4:** Fit indices for the multi-trait/multi-method models.

								90% Cl	
Model	df	χ^2^	Δχ^2^	AIC	CFI	TLI	RMSEA	Lower	Upper
CTCM	358	703.682**		917.682	0.908	0.889	0.058	0.051	0.064
CTUM	359	696.907**	−6.775**	908.907	0.911	0.862	0.057	0.051	0.063
CTPCM	359	706.281**	2.599	918.281	0.908	0.889	0.058	0.052	0.064
PCTCM	370	754.871**	51.189**	962.871	0.896	0.874	0.061	0.055	0.068
UTCM	370	628.323**	−75.359**	836.323	0.929	0.915	0.051	0.044	0.057
NTCM	391	889.231**	185.549**	1,037.213	0.868	0.853	0.066	0.061	0.072

*CTCM, correlated trait/correlated methods; CTUM, correlated traits/uncorrelated methods; CTPCM, correlated traits/perfectly correlated methods; PCTCM, perfectly correlated traits/correlated methods; Uncorrelated traits/correlated methods; NTCM, no traits/correlated methods.*

**p significant at 0.01.

### Discriminant Validity and Convergent Validity

Examining the extent to which the independent measures of the same trait are correlated provides an indication of convergent validity. A significant difference between a model where the traits are specified and one where the traits are not specified provides evidence of convergent validity. Evidence of convergent validity is calculated by assessing the Δχ^2^ between the CTCM model and the NTCM model (Cresswell and Eklund, 2006a). Discriminant validity is supported by traits and methods with low correlations between independent measures of different subscales providing evidence. Discriminant validity of traits is manifested by significant Δχ^2^ between the CTCM model and the PCTCM. In the current study discriminant validity of method and traits are provided by a significant difference in χ^2^ between: (1) the CTCM and PCTCM models, and (2) the PCTCM and CTPCM models; as well as non-significant difference between: (1) CTCM and UTCM models, and (2) the CTCM and CTUM models (Byrne, 1994b). The comparison of the CTCM and CTUM models tests whether the methods are correlated and determines whether the traits are related; a non-significant difference provides an indication of discriminant validity.

The comparison of the MTMM models with the baseline CTCM model for the purpose of evaluating convergent and discriminant validity were conducted using the Δχ^2^ tests. The Δχ^2^ and AIC values for each of the models are reported in Table 4. Evidence of trait and method discriminant validity is supported by a statistically significant Δχ^2^ (Δχ^2^ = 51.189, *p* < 0.001) between CTCM (χ^2^ = 703.682) and PCTCM (χ^2^ = 754.871) as well as a statistically significant Δχ^2^ (Δχ^2^ = 48.590, *p* < 0.001) between CTPCM (χ^2^ = 706.281) and PCTCM. This is reinforced by the large increase in AIC between CTCM (AIC = 917.682) and PCTCM (AIC = 962.871) as well as CTPCM (AIC = 918.281) and PCTCM. The significant Δχ^2^ between CTCM and CTPCM models supports discriminant validity between methods. The difference between CTCM and PCTCM provides support for discriminant validity between traits. The significant Δχ^2^ between CTCM and NTCM provides evidence of convergent validity. However, this is not supported by the significant difference between CTCM and CTUM (Δχ^2^ = −6.775, *p* < 0.001) and CTCM and UTCM (Δχ^2^ = −75.359, *p* < 0.001).

Campbell and Fiske (1959) suggested that the evaluation of patterns of the correlations within the MTMM matrix could provide evidence of convergent and discriminant validity. Marsh et al. (2002) highlight that MTMM evaluation of construct validity through SEM are useful because data factor structures can be evaluated while also appropriately correcting constructs for measurement error. The correlations between the trait variables (i.e., reduced accomplishment, exhaustion, sport devaluation) represent the discriminant validity between the different traits. These correlations should not be too high (*r* > 0.70; Eid et al., 2008). Correlations between reduced accomplishment and sport devaluation were below the *r* > 0.70 threshold indicating discriminant validity. Reduced accomplishment and sport devaluation correlation was statistically significant (factor *r* = 0.634, *p* < 0.001). Exhaustion shows low correlations to sport devaluation (factor *r* = 0.153, *p* = 0.100) and reduced accomplishment (factor *r* = 0.139, *p* = 0.116). This indicates that the three traits (i.e., reduced accomplishment, exhaustion, sport devaluation) have high discriminant validity and are justified as different constructs in the scale.

The correlations between trait-specific method factors determine the generalizability of method effects across traits (i.e., teammate reduced accomplishment, TRA; teammate exhaustion, TE; teammate sport devaluation, TSD). The correlation between TRA and TSD was above the *r* > 0.70 threshold (*r* = 0.879, *p* < 0.001). TE shows good discriminant validity with TRA (*r* = 0.442, *p* < 0.001) and TSD (*r* = 0.583, *p* < 0.001). These correlations specify how strongly an over- or underestimation of one of the trait-specific method factors is related to the over- or underestimation on the other trait-specific method factor of the same method. Correlations between TBQ methods and ABQ methods were also conducted ranging from *r*s 0.275–0.750 (see Table 3). Although the athlete exhaustion and teammate exhaustion correlation was above the *r* > 0.70 threshold, Raedeke et al. (2013) found similar findings acceptable. Furthermore, Marsh et al. (2002) would consider the size of these correlations relative to the convergent correlations to be well within the tolerable range. The factor loadings are shown in Tables 5A–C, offering further support for the validation of the TBQ. Items 1, 5, and 14 (teammate reduced accomplishment) loaded well on to trait (factor loading ranged from 0.187 to 0.233) and method (factor loading ranged from 0.450 to 0.644). Results related to items 7 and 13 (teammate reduced accomplishment) indicated low loading onto trait (factor loading ranged from 0.027 to 0.072) and high loading on to method (factor loading ranged from 0.699 to 0.791). The results emphasize the high loading of the teammate exhaustion items on to the trait (factor loading ranged from 0.511 to 0.773) and the method (factor loading ranged from 0.354 to 0.430). The results also highlight the high loading of four of the sport devaluation items (i.e., 3, 6, 9, and 11) on the trait (factor loading ranged from 0.246 to 0.316) and the method (factor loading ranged from 0.477 to 0.661). Item 15 (teammate reduced accomplishment) results highlighted low loading on trait (0.097) and high on to method (0.673). Therefore, the MTMM provides support for the convergent and discriminant validity of the subscales within the TBQ and ABQ.

**TABLE 5A:** Standardized trait and method-specific factor loading in correlated trait/correlated methods (CTCM) Model (part 1).

Reduced accomplishment					
	T1-RA	T2-E	T3-SD	ABQ	TBQ
**ABQ items**					
1	0.534**			0.027	
5	0.648**			0.103	
7	0.699**			0.372**	
13	0.498**			0.397**	
14	0.661**			0.156*	
**TBQ items**					
1	0.233**				0.450**
5	0.187*				0.642**
7	0.027				0.699**
13	0.072				0.791**
14	0.225**				0.460**

TBQ, team burnout questionnaire; ABQ, athlete burnout questionnaire; TI-RA, trait one reduced accomplishment; T2-E, trait two exhaustion; T3-SD, trait three sport devaluation. The symbol ** represents significance at 0.01.

**TABLE 5B:** Standardized trait and method-specific factor loading in correlated trait/correlated methods (CTCM) Model (part 2)

Exhaustion					
	T1-RA	T2-E	T3-SD	ABQ	TBQ
**ABQ items**					
2		0.410**		0.398**	
4		0.472**		0.433**	
8		0.559**		0.578**	
10		0.525**		0.579**	
12		0.453**		0.648**	
**TBQ items**					
2		0.511**			0.399**
4		0.537**			0.430**
8		0.660**			0.395**
10		0.773**			0.354**
12		0.658**			0.405**

TBQ, team burnout questionnaire; ABQ, athlete burnout questionnaire; TI-RA, trait one reduced accomplishment; T2-E, trait two exhaustion; T3-SD, trait three sport devaluation. The symbol ** represents significance at 0.01.

**TABLE 5C:** Standardized trait and method-specific factor loading in correlated trait/correlated methods (CTCM) Model (part 3).

Sport devaluation					
	T1-RA	T2-E	T3-SD	ABQ	TBQ
**ABQ items**					
3			0.325**	0.276**	
6			0.772**	0.284**	
9			0.673**	0.433**	
11			0.668**	0.235**	
15			0.313**	0.359**	
**TBQ items**					
3			0.273**		0.477**
6			0.246**		0.613**
9			0.300**		0.661**
11			0.316**		0.535**
15			0.097		0.673**

TBQ, team burnout questionnaire; ABQ, athlete burnout questionnaire; TI-RA, trait one reduced accomplishment; T2-E, trait two exhaustion; T3-SD, trait three sport devaluation. The symbol ** represents significance at 0.01.

## Discussion

The purpose of the present study was to validate a measure of athletes’ perceptions of their teammates’ burnout. Central to this aim was an assessment of the factorial, convergent, and discriminant validity of the factors comprising the ABQ and TBQ (i.e., exhaustion, reduced accomplishment, and sport devaluation). The factorial validity of the ABQ and the TBQ were supported through the CFAs. The CFA of the ABQ supports the three-factor solution (Raedeke and Smith, 2001) and good model fit was found for a first-order and second-order model as seen in previous research (Isoard-Gautheur et al., 2010; Gerber et al., 2018). The CFA for the TBQ also indicated good fit, however, further research is required to support the three-factors solution for teammate burnout. Despite the findings suggesting second-order model fit with the empirical data, Gerber et al. (2018) highlighted the difficulty of grouping the subscales of ABQ (and TBQ) under the same label as this contradicts the recommendation of the MBI-GS manual which suggests they should be measured independently and not combined (Maslach et al., 1986, 1996).

Although there were limitations observed in both measures, the findings of the MTMM analysis support the discriminant and convergent validity of the ABQ and TBQ in a sample of team sport athletes. Specifically, the correlations of the equivalent subscales across the two burnout measures (i.e., reduced accomplishment and teammate reduced accomplishment) are high, indicating that both scales had good convergent validity. However, this could be explained by three items of teammate reduced accomplishment loading well on the trait but not the method. Furthermore, the correlations between equivalent subscales were higher than for non-matching subscales; although, there was a stronger correlation between teammate reduced accomplishment (i.e., perception of teammates) and sport devaluation (i.e., self) compared to teammate reduced accomplishment and reduced accomplishment. Furthermore, the within method correlation for both ABQ and the TBQ subscales were strongly correlated. High internal discriminant validity was also observed between the methods. As the loading of the TBQ items onto the subscales of the TBQ suggest sufficient discriminant validity of the TBQ as a measure for assessing an individual athlete’s perceptions of teammates’ burnout (Eid et al., 2003).

Whilst these findings support the convergent and divergent validity of the TBQ and ABQ, it is important that future research replicates the present study using varied samples (Raedeke et al., 2013) in order to validate the TBQ with athletes from more diverse competitive sport environments. For example, studies testing the utility of the TBQ within a range of team sport settings may determine the measure’s effectiveness in assessing athletes’ perceptions of their teammates’ burnout across age groups (e.g., youth sport) and levels of competition (e.g., elite; Davis et al., 2019a). Further, research may also wish to consider the size of teams being assessed. Across competitive sport, the number of the athletes on a team varies from two in doubles racquet sports (e.g., tennis) to squads comprised of more than forty-five players (e.g., American football). In consideration of the degree of intimacy and frequency of interactions as a function of team size, it is possible that the number of individuals on a team may influence the athlete’s perception of their teammates’ burnout and the accuracy of this perception. As such, future research should explore the utility of the TBQ across team settings comprised of various numbers of individuals, as well as consider the nature of the interactions within the team and possible sub-groups (e.g., offense vs. defense in American Football).

An increasing number of athletes are reporting symptoms of burnout (Madigan et al., 2022), and previous research indicates that an individual’s contextual environment (González-Morales et al., 2012) and social support (Lu et al., 2016; Simons and Bird, 2022) can influence levels of athlete burnout and wellbeing. As such, the availability of the TBQ to assess the potential influence an athlete’s perceptions of his/her teammates’ burnout on the individual athlete (Appleby et al., 2018) is a timely contribution to burnout research. The development of the TBQ can promote researchers’ examination of the possible antecedents to athlete burnout (Gustafsson et al., 2011); it can advance research beyond viewing burnout as an individual phenomenon (Madigan et al., 2021), and widen appreciation of the social and organizational context of sport (DeFreese et al., 2021). The TBQ may assist in elucidating the mechanisms of burnout at a team level; for example, burnout contagion may spread through interpersonal emotion regulation (Davis et al., 2018b; Tamminen et al., 2019) or communication between teammates and with coaches (Davis et al., 2018a,2019b).

From an applied perspective, sport psychologists working with teams could use the TBQ to gauge perceptions of burnout within a team and facilitate the development of targeted interventions to improve athlete well-being. The TBQ may also be incorporated into studies aiming to elucidate the factors influencing athletes’ contextual performance environment and interpersonal relationships (e.g., coaches). For example, previous research has suggested that basic psychological needs mediate the relationship between perfectionism and athlete burnout (Jowett et al., 2016). Future studies may examine whether an athlete’s perception of their teammates’ burnout mediates the relationship between needs thwarting or needs satisfaction behaviors and their own burnout.

The present study advances the potential for increasing understanding of burnout within sports teams; however, it is not without limitations. First, the TBQ was validated with a sample of adult athletes, therefore its utility with younger age groups remains uncertain. As burnout is on the rise in adolescent and elite athletes (Gustafsson et al., 2007, 2008), it is recommended that the TBQ is validated for use with these populations. The TBQ currently considers the team as a collective, it does not reflect potential variability in burnout across the members comprising the team. Moreover, the size of the team and the degree of interactions between teammates may influence individuals’ perceptions of the team as a whole. That said, the TBQ is designed to capture individual athletes’ perception of their social environment at the team level similar to measures of other social constructs (e.g., group cohesion). Second, it is important to note that most athletes in the current study perceived their teammates as healthy and expressing low levels of burnout. Although, burnout research has predominantly investigated athletes reporting relatively low levels of burnout (Gould and Whitley, 2009; Gustafsson et al., 2011); to alleviate potentially confounding measurement issues, previous research suggests considering the “healthy worker” effect (Chowdhury et al., 2017). In particular, burnout research has predominantly been undertaken with athletes that are healthy enough to maintain participation in sport, comprehensive study of burnout would benefit from extended sampling that includes those athletes that have dropped out of sport as a result of burnout severity that precludes involvement in sport (Gustafsson et al., 2011). To advance sport psychology research and practice, future studies should attempt to examine samples of athletes who perceive their teammates to be experiencing higher levels of burnout. Finally, the cross-sectional research design used in the present study does not permit the examination of the changes over time or allow for the long-term impact of these perceptions to be investigated (DeFreese and Smith, 2020). For example, future studies may aim to map how potential crossover processes (e.g., interpersonal emotion regulation; Tamminen and Crocker, 2013) develop within teams over time and are associated with burnout at the team level.

In summary, the present study sought to determine the preliminary validity of the TBQ; it reports satisfactory discriminant and convergent validity of the ABQ and the TBQ. As such, the findings indicate that researchers should be confident in using the ABQ and the TBQ in team sports contexts. In particular, the availability of a validated quantitative measure of a social factor associated with athlete burnout addresses a limitation of previous research and can increase understanding of the athlete’s experience of their social environment (Pacewicz et al., 2019; DeFreese et al., 2021). Wider study and use of the TBQ can contribute to the advancement of sport psychology research and applied practice, with the aim of promoting positive social environments to optimize athletes’ performance and wellbeing.

## Data Availability Statement

The raw data supporting the conclusions of this article will be made available by the authors, without undue reservation.

## Ethics Statement

The studies involving human participants were reviewed and approved by Department of Sport, Exercise, and Rehabilitation Ethics Board at Northumbria University, Newcastle, United Kingdom. The patients/participants provided their written informed consent to participate in this study.

## Author Contributions

RA was involved in designing the study, collecting the data, analyzing the data, and wrote the manuscript. PD, LD, and WV were involved in designing the study, analyzing the data, and wrote the manuscript. AS was involved in analyzing the data and wrote the manuscript. All authors contributed to the article and approved the submitted version.

## Conflict of Interest

The authors declare that the research was conducted in the absence of any commercial or financial relationships that could be construed as a potential conflict of interest.

## Publisher’s Note

All claims expressed in this article are solely those of the authors and do not necessarily represent those of their affiliated organizations, or those of the publisher, the editors and the reviewers. Any product that may be evaluated in this article, or claim that may be made by its manufacturer, is not guaranteed or endorsed by the publisher.
